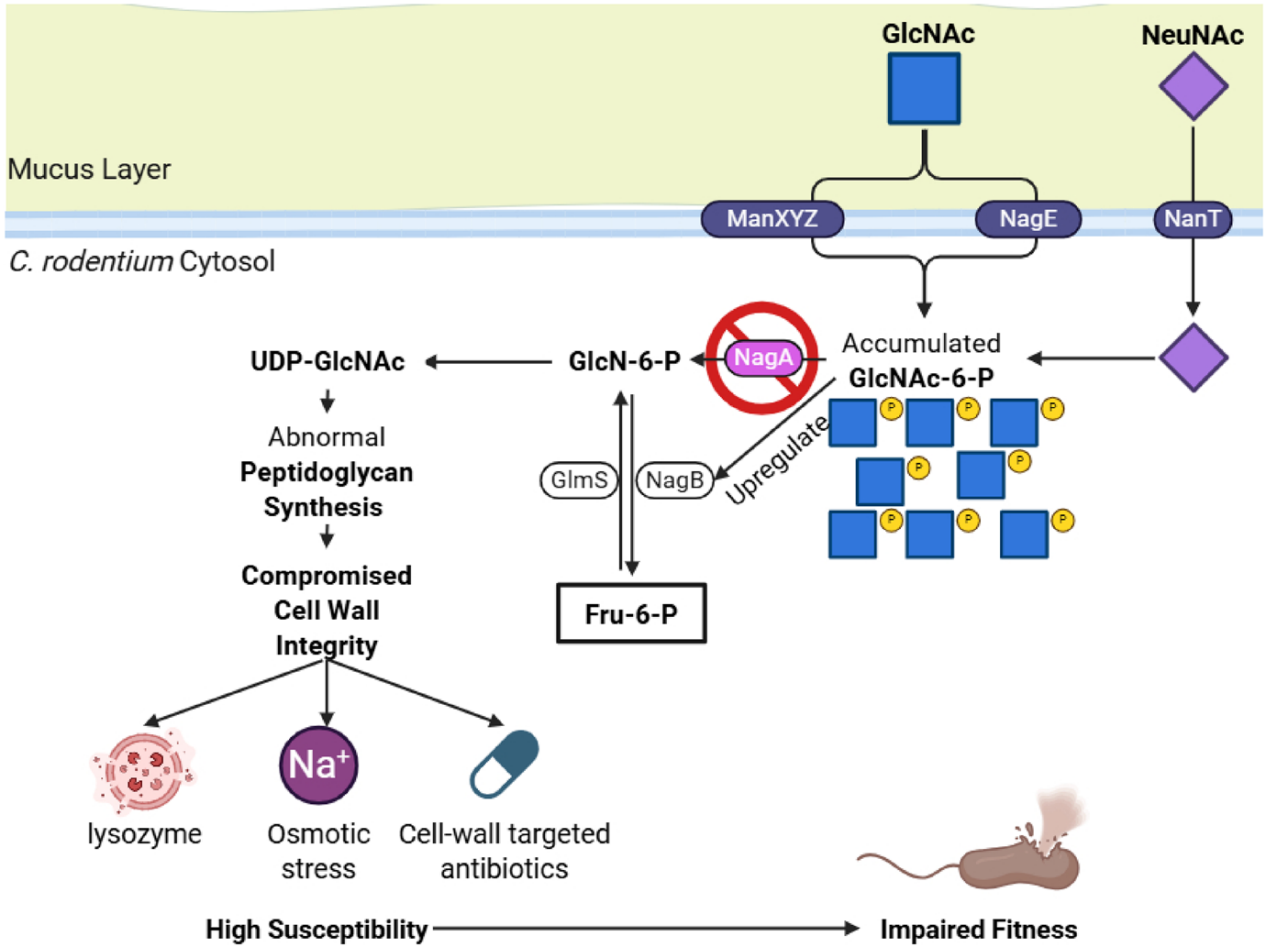# Poster Session I - Poster of Distinction I - A21 DISRUPTION OF MUCIN-DERIVED SUGAR METABOLISM PATHWAYS IMPAIRS ENTERIC PATHOGEN *CITROBACTER RODENTIUM* COLONIZATION

**DOI:** 10.1093/jcag/gwaf042.021

**Published:** 2026-02-13

**Authors:** Z C Huang, M A Mslati, C Ma, H Yang, Q Liang, S Crowley, A Gilliland, R Dyer, I Ng, H Yu, B Vallance

**Affiliations:** Experimental Medicine, The University of British Columbia, Vancouver, BC, Canada; Experimental Medicine, The University of British Columbia, Vancouver, BC, Canada; Experimental Medicine, The University of British Columbia, Vancouver, BC, Canada; Experimental Medicine, The University of British Columbia, Vancouver, BC, Canada; Experimental Medicine, The University of British Columbia, Vancouver, BC, Canada; Experimental Medicine, The University of British Columbia, Vancouver, BC, Canada; Experimental Medicine, The University of British Columbia, Vancouver, BC, Canada; Experimental Medicine, The University of British Columbia, Vancouver, BC, Canada; Experimental Medicine, The University of British Columbia, Vancouver, BC, Canada; The University of Kansas Medical Center, Kansas City, KS; Experimental Medicine, The University of British Columbia, Vancouver, BC, Canada

## Abstract

**Background:**

Enteric bacterial pathogens within the *Enterobacteriaceae* family, including *Escherichia coli* and *Salmonella* species, can cause acute gastroenteritis in humans. In the competitive gastrointestinal environment, these pathogens depend on specific metabolic adaptations to establish infections. The intestinal mucus barrier, composed primarily of the mucin Muc2, protects the epithelium while also serving as a nutrient reservoir rich in host-derived monosaccharides such as N-acetylglucosamine (GlcNAc) and N-acetylneuraminic acid (NeuNAc). How these mucus-derived sugars are metabolized during infection and how dysregulation of these pathways affects bacterial pathogen fitness remain poorly understood.

**Aims:**

We sought to determine how *Enterobacteriaceae* exploit mucin-derived sugars for colonization and whether disrupting these pathways impairs their fitness. *Citrobacter rodentium*, a murine-specific enteric pathogen, was used as a model system and was hypothesized to be able to exploit GlcNAc and NeuNAc to fuel their pathogenesis.

**Methods:**

*C. rodentium* mutants were generated, including Δ*nagA* lacking GlcNAc-6P deacetylase, its complemented strain, and Δ*mana* lacking GlcNAc/NeuNAc import systems. Colonization and tissue pathology were examined in C57BL/6J mice. Intracellular GlcNAc-6P was quantified by the Morgan–Elson assay. Transcriptional responses were analyzed by RT-qPCR. Bacterial susceptibility to cell wall stress was tested using lysozyme, osmotic, and antibiotic challenges.

**Results:**

Deletion of *nagA* severely attenuated colonization and pathology in mice, while Δ*mana* remained fully infective, indicating that virulence loss was not solely due to nutrient deprivation. Δ*nagA* accumulated GlcNAc-6P, grew poorly *in vitro*, upregulated *nagB* transcription, suggesting a disrupted glucosamine-6-phosphate (GlcN-6P) synthesis. Functionally, Δ*nagA* exhibited increased sensitivity to lysozyme, osmotic stress, and cell wall-targeting antibiotics, indicating that cell wall integrity was compromised.

**Conclusions:**

Disrupting *nagA* in *C. rodentium* causes GlcNAc-6P accumulation and disrupted GlcN-6P synthesis, compromising cell wall integrity and *in vivo* fitness. As these amino sugar catabolic pathways are conserved across *Enterobacteriaceae*, this work identifies a previously unrecognized metabolic vulnerability that could be exploited to weaken enteric pathogens such as pathogenic *E. coli* and *Salmonella*. Targeting sugar-phosphate stress pathways offers a promising approach to limit bacterial infections of the gastrointestinal mucosa.

**Funding Agencies:**

CIHRNatural Sciences and Engineering Research Council of Canada